# Genetic Testing Consumers in Italy: A Preliminary Investigation of the Socio-Demographic Profile, Health-Related Habits, and Decision Purposes

**DOI:** 10.3389/fpubh.2020.00511

**Published:** 2020-10-08

**Authors:** Serena Oliveri, Giulia Marton, Laura Vergani, Ilaria Cutica, Alessandra Gorini, Francesca Spinella, Gabriella Pravettoni

**Affiliations:** ^1^Applied Research Division for Cognitive and Psychological Science, European Institute of Oncology, Istituto Europeo di Oncologia, Istituto di Ricovero e Cura a Carattere Scientifico, Milan, Italy; ^2^Department of Oncology and Hemato-Oncology, University of Milan, Milan, Italy; ^3^GENOMA Molecular Genetics Laboratory, Rome, Italy

**Keywords:** direct to consumer genetic testing, consumers' socio-demographic profile, public health, decision making, education in genetics

## Abstract

**Aim of the study:** Genetic testing is becoming increasingly common in clinical practice and health management; nonetheless, little is known about how the population approaches genetic services through private companies. Our study aims to describe socio-demographic aspects, health-related habits, and overall beliefs and knowledge about genetic risk and testing in a population of Italian citizens who decided to undergo a genetic examination through a private genetic company.

**Study design:** A sample of 152 clients from an Italian private genetic company completed an *ad-hoc* survey from September 2016 to February 2018, addressing socio-demographic data, health habits, psycho-physic condition, perceived utility of genetic results, decision purposes about data sharing, and behavioral changes after results.

**Results:** Participants (mean age 42.4) were predominantly female (82.2%) and were overall well-educated. Their main source of information were physicians (77%), and 41.1% entrusted the management of results to the same. Thirty-eight percentage underwent genetic analysis for cancer predisposition, 31.3% for fertility problems, 24% for dietary or intolerance issues in the period of enrolment. More than half of them (62.7%) reported a family history of the disease, and overall 69% had a current or past experience with a disease. Clients perceived the genetic screening as useful to adopt behaviors that may prevent disease onset (37.7%), to know their “real health status” (27.4%), and to adopt health-related behaviors (23.3%). 62.8% claimed they were motivated to change behaviors after results (healthier diet, practice exercise, medical checks), and they wanted to share results with their physician and family members.

**Discussion/Conclusion:** The overview of consumers' profiles in Italy and other European countries can contribute to tailoring and regulating genetic services in a way that could be efficient in terms of healthy choices, behaviors, and health resource expenditures for the general public.

## Introduction

Before the sequencing of the human genome, which refers to the individual's entire genetic makeup, the general public had little reason to understand human genetics research and freely decide to undergo genetic testing for health-related purposes, since the science was limited and had little impact on most people's daily lives. That situation changed, since modern research has revealed genetic associations with the leading causes of mortality, including cancer, diabetes, and heart disease (1, 2), making genetic research relevant for nearly everyone. Nowadays, genetic testing constitutes an efficient preventive and diagnostic support (3). Furthermore, genomic science now addresses genes and genetic materials as a dynamic system, revealing how genes work and interact with non-genetic factors, including the environment, and lifestyle experiences (4–6). While supporters of Direct to Consumer Genetic Testing (DTC-GT) argue that people should be completely free to decide to purchase a genetic service based on their personal needs and to manage their genetic risk information, for other experts, GT utility must be carefully evaluated case by case (7, 8). However, the current perspective is that gathering genetic risk information is not harmful but, on the contrary, could help people to make more informed decisions about their health, and GT users can be empowered in sharing decisions with their referred physicians (9–11).

Differently from the US social and cultural context, the DTC-GT was rather unknown in Italy until the first decade of the 2000s, since genetic tests have stayed mainly in the hands of experts. The Italian population seems to be far behind in terms of awareness of the opportunity to know their genetic profile for health purposes, and the implication of such information (12). Results from Oliveri et al. (12) highlighted the Italian lay people's interest in using DTC-GT, along with the need for a health care professional to help them interpret the results. A similar context and tendency seem to characterize other European populations, such as in Greece (13, 14), the Netherlands (15), Germany (16), or Switzerland (17). Most of the articles available in the literature concerning DTC-GT consumers are focused on countries outside the European Union (the US in particular) and investigated perspectives in laypeople or highly educated groups of graduate students who had never purchased a DTC-GT (18, 19). Few studies reported outcomes on actual consumers in Europe. Su et al. (20) collected stories of customers from a European context (countries not specified) who purchased DTC-GT for themselves and/or for their families, retrieved from non-genetic company websites as well as from DTC-GT companies' websites. The contribution focuses on consumers' motivations and expectations for having purchased genetic testing through a private laboratory, with a very short description of their socio-demographic profile, that was based on the personal information provided by customers in their stories (such as that they were highly specialized professionals).

Vayena et al. (17) reported attitudes, motivations, and self-reported impacts of results in a sample of DTC-GT consumers (40 students from two higher education institutions in Zurich, Switzerland) and provided a valuable hypothesis of why DTC-GTs are not commercialized in Europe as much as in the US or UK, or why there is little knowledge of their existence. Participants were all scientists of the Swiss Federal Institute of Technology (ETH) and of the University of Zurich in Switzerland, thus the sample was not representative or comparable with the broader population.

Recently, Wöhlke et al. (21) contributed to profiling actual DTC-GT consumers from two European countries: Germany and Italy. This investigation reports that Italian DTC-GT users gave higher importance to the genetic risk information in terms of prevention for themselves and their families, and believed that they could counter-balance a genetic predisposition with preventive measures more than Germans users. Another interesting cultural difference concerned the perception of genetic information as providing certainty, which was supported by about three-quarters of German participants and only a few Italian responders. Thus, Italians seem not to expect to receive a certainty from GT results about their future health condition, but consider them useful for gathering data that allow them to actively face the risk.

In Belgium, Italy, and the UK there is no specific legislation that forbids or regulates the provision of DTC-GT, while in France, Germany, Portugal, and Switzerland there is a specific legislation that dictates that genetic tests can only be carried out by a medical doctor after the provision of sufficient information and appropriate genetic counseling (22, 23). Nevertheless, to develop specific regulations and policies, it is necessary to know more about the profile, and motivation of people who decide to purchase a genetic test from private companies (24, 25). Socio-demographic aspects, health habits, perceived beliefs, and literacy may impact the attitudes, interests, and understanding toward genetic services (26, 27).

This contribution aims to provide a preliminary description of GT consumers' in Italy, sounding out their socio-demographic aspects, health-related habits, beliefs, and knowledge about genetic risks and testing.

## Materials and Methods

Participants were recruited via GenomaLab–Molecular Genetics Laboratory, in Rome and Milan. GenomaLab is a private genetic company, that offers a wide range of panels of genetic analysis for disease susceptibility/risk. It provides their clients with a focused and personalized counseling. Clients who underwent genetic testing for disease carriers, infertility problems, disease risk susceptibility, food intolerances, or wellness were included in our survey. Clients who required genetic testing during pregnancy or for Medically Assisted Procreation (MAP) (PrenatalSafe, PrenatalScreen, Preimplantation genetic diagnosis, etc.) were excluded since they belong to an area accountable for being treated in a dedicated study. All participants signed informed consent forms and completed the survey before undergoing GT, in the waiting room of the Lab, through a paper-and-pencil version administered by the main experimenter of this study, or before going to the Lab for blood sampling, through the Survey Monkey platform online. Recruitment started in February 2016 and ended in September 2018. As an incentive, participants were given a discount on the cost of their genetic tests. The price range for testing in this Lab varies from a minimum of 80–100 euros for genetic lifestyle analysis, specific intolerances, or one specific disease, to 450–500 euros for a predictive package analysis for a series of diseases (e.g., different types of cancer). A total of 152 participants agreed to complete the questionnaire. The response rate was 48% of 317 clients who underwent genetic testing in the period of recruitment.

The research protocol was approved by the Institutional Review Board of the University of Milan, the principal coordination center of the survey, and by the Center for Research Ethics and Bioethics, University of Uppsala, coordinator of the Mind the Risk project (see funding declaration). The study was conducted according to the Helsinki declaration.

*Materials*. A structured and self-administered *ad hoc* questionnaire (see Supplementary Material for the questionnaire) was created to assess the domains described in Table 1.

**Table 1:** Questionnaire for the assessment of consumers' profile.

**Assessed domain**	**Items**	**Kind of questions/answers**
Socio-demographic	What's your gender/age/relationship status/number of children/educational level/current employment?	Multiple-choice questions
Lifestyle, health-related habits	- Which kind of diet do you follow, mainly?; - How are your sleep habits?; - Do you undergo medical checkups?; - International physical activity questionnaire–short form (28)	Multiple-choice questions and standardized scales
Physical condition and disease history	- Body max index calculation; - Do you or have you suffered anxiety, depression, or sleep disorders? - Have you suffered/are currently suffering from specific diseases?; - In your family history, is there a relevant experience of illness? - Do you have a family history of genetically/inherited diseases?	“yes”, “no”, “I do not know” answer options
Users motivation for genetic testing and the genetic analysis performed	- What genetic service did you buy? - What is the reason you decided to undergo a genetic analysis?	Open-ended questions
Management of genetic results	- How are you in contact with Genoma Group? (details of results management in the answers options)	Multiple-choice questions
Risk behaviors	- Do you smoke (cigarettes, cigars, pipe, other)? If yes, about how many cigarettes, cigars, pipes, or other do (did) you smoke in a typical day?; - How often do you…[List of 7 dietary risk behaviors: fast food; junk food happy hour; sandwiches; fried food; dessert/cake/chocolate; drink soda; drink alcohol]	- “yes”, “no”, “in the past” answer options + the number of cigarettes etc.; - 5-point Likert scale, from 0 “never” to 4 “daily”
Genetic information source and knowledge	- How did you learn about the opportunity to undergo a genetic analysis? - What knowledge do you have about genetic risk and genetic screening? - Did you receive counseling before undergoing genetic analysis? Specify which professional.	Multiple-choice questions
Perceived utility of genetic results	What kind of utility do you think genetic screening may have for your health?	Multiple-choice question
Decision purposes for data sharing and behavioral changes.	- Do you think your lifestyle will change after receiving the results of the genetic analysis? “Read each item and decide which health behaviors can reflect your lifestyle changes,” multiple choices; - After receiving the results of the genetic analysis, do you think it is better to share the results with.?	Multiple-choice questions

The questionnaire took around 45 min to complete.

### Data Analysis

Descriptive statistics (frequencies and percentages) were calculated on raw data to report participants' socio-demographic characteristics, their health-related habits, their psycho-physic condition, and their motivation toward genetic testing. Contingency tables and Chi-Square tests were then performed to compare subgroups distinguished based on of the above-mentioned variables and the level of consumers' perceived knowledge about genetics. Expected values and residuals in every box were calculated to verify if a specific group gave a significantly higher or lower rate of response (observed values) to certain items, compared to the percentage expected and calculated on the number of subjects recruited. Analyses were performed with SPSS (25.0, IBM, USA, 2014).

## Results

Table 2 reports the socio-demographic characteristics of the participants.

**Table 2:** Socio-demographic description of participants.

	**Number**	**Percent**
**Gender**		
Male	27	17.8
Female	125	82.2
**Marital status**		
Single	21	13.9
In a relationship	11	7.3
Cohabitant	25	16.5
Married	89	59
Divorced	4	2.6
Widowed	1	0.7
**Offspring**		
No	74	49,3
Yes	76	50,7
**Education**		
Primary school	2	1.3
Secondary school	7	4.6
High school	65	42.8
University	62	40.8
Post-university	16	10.5
**Occupation**		
Students	14	9.2
Unemployed	9	5.9
Housewife	10	6.6
Blue collar	3	2.0
White collar	64	42
Freelance	41	27
Manager	1	0.7
Retired	10	6.6
	**Mean**	**SD**
Age	42.4	13

Participant's mean age was 42.4 years (min. 11, max. 76; SD = 13) and most of them were female (82.2%). More than half of the population was married (58.9%) (followed by co-habitants 16.5% and single 13.9%); 49.3% did not have any child whereas 50.7% had one or more than one child. The overall sample was well-educated: 42.8% completed high-school while more than 51% had an academic education (40.8% University, 10.5% postgraduate education). They were white-collar workers (42%) or freelance workers (27%) mainly (details in Table 2).

The mean score for BMI was 23.2, indicating an overall normal weight range.

### Lifestyle, Health-Related Habits, and Psycho-Physic Condition

Ninety-three percentage of participants declared to follow a Mediterranean diet (vegetables, meat, fish, and pasta), 44.7% declared to usually practice moderate physical activity, 32.2% light physical activity, and 23% vigorous physical activity. Their sleep quality was generally poor: 44.3% answered that they woke up very early in the morning and could not continue to sleep during the night, or they fall asleep often even during the day and wake up often during the night. Just 22.8% of the sample fell asleep easily and had restorative sleep.

**Figure 1:**
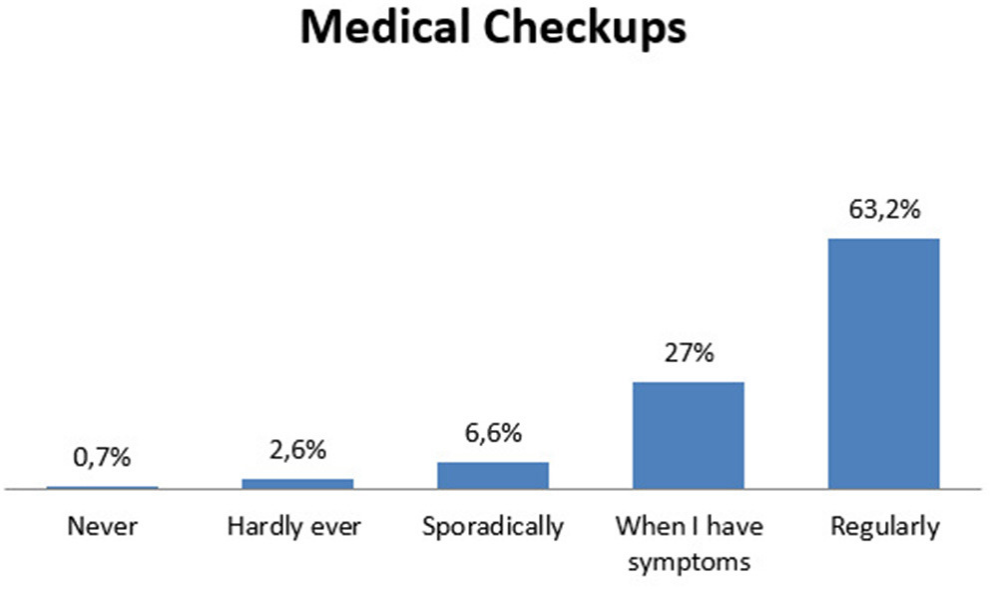
Clients' habits in medical checkups.

Among participants, 40.7% had past or present psychological problems (sleep disorders, anxiety, depression). They were also asked about the frequency of their medical checkups: the majority underwent medical checkups regularly (63.2%), as described in Figure 1.

As described in Table 3, 34.9% of the sample had a specific disease at the moment of genetic analysis, while 34.2% had suffered from a specific disease in the past. More interestingly, a large number of clients (62.7%) reported a family history of the disease, but only 24.3% recognized that a genetic predisposition might run in the family, whereas 37.5% claimed the opposite and 38.2% did not know.

**Table 3:** Consumers' disease background.

**Disease History**			
	**No**	**Yes**	**“I don't know”**
Past diseases	65.8% (98)	34.2% (51)	–
Current diseases	65.1% (99)	34.9% (53)	–
Family diseases	27.3% (41)	62.7% (94)	10% (15)
Genetic diseases	37.5% (57)	24.3% (37)	38.2% (58)

### Users Motivation for Genetic Testing, the Genetic Analysis Performed and Management of Genetic Results

**Figure 2:**
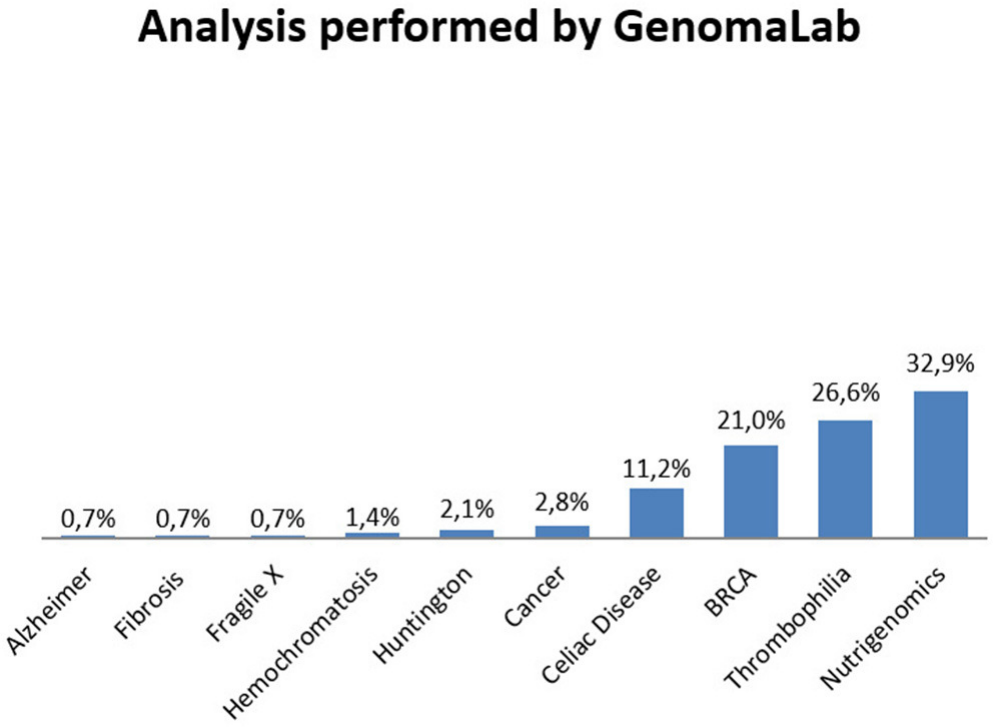
Analysis performed by GenomaLab in the period of recruitment.

Thirty-eight percentage of participants underwent the genetic test to know about their predisposition to cancer (BRCA 1 and 2 testing or susceptibility to cancer in general, Oncoscreening); 31.3% required analysis for fertility problems or to prevent genetic transmission to the offspring (Celiac Disease, X Fragile, Cystic Fibrosis); 24% for dietary or intolerance investigation; 6.7% to test their predisposition to other diseases such as Thrombophilia, Huntington, and Alzheimer's disease. The genetic analysis performed by GenomaLab on their clients is represented in Figure 2.

**Figure 3:**
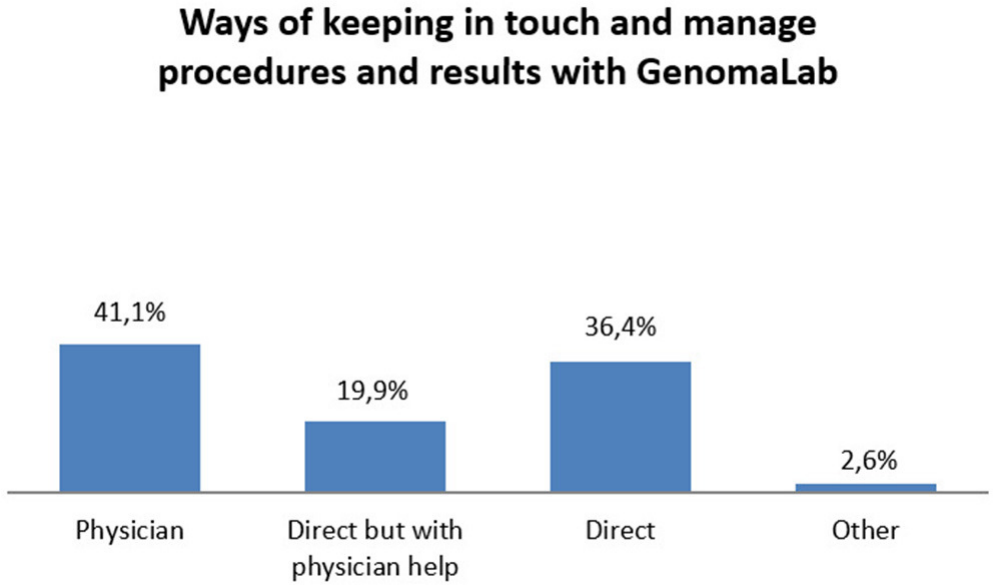
Contact with GenomaLab for analysis and result management.

The main way that participants learned about private genetic testing and GenomaLab was through their family physician or specialist (gynecologists, geneticists, etc.) (77%), followed by mass media (9.2%), relatives (7.9%), and friends (7.2%) in almost equal extent. Clients claimed that their preferred way of keeping in touch and to manage procedures and results was through their physicians (41.1%), but 36.4% of them decided to directly keep in contact with the genetic lab, handling the results without the physicians as a mediator (see Figure 3).

Pearson Chi-square test showed a significant association between having learned about and having been referred to GenomaLab through a physician and the decision of handling the procedures and communication with the GenomaLab through the physician as a mediator, *X*^2^ (*N* = 151) = 22.349, *p* = 0.000.

### Risk Behaviors

19.3% of participants were current smokers at the moment of enrollment (a mean of 8.4 cigarettes per day), while 24% of the sample stated that they were former smokers. The mean scores for dietary risk behaviors were 13 (max total scoring was 28, representing daily unhealthy food intake).

### Genetic Risk/Screening Information Source, Perceived Utility of Genetic Results, Decision Purposes About Data Sharing and Behavioral Changes

**Figure 4:**
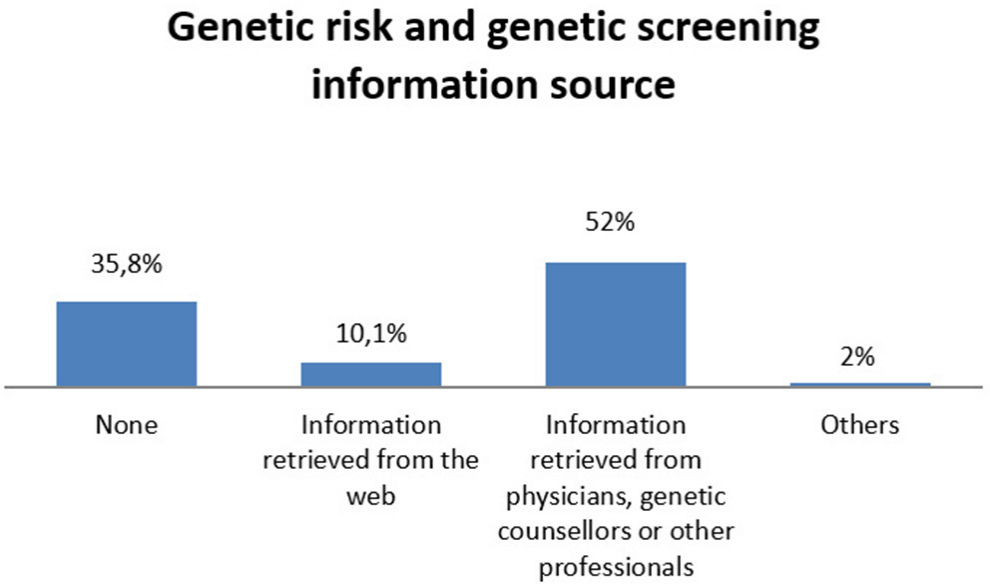
Sources where consumers collected information for genetic risk and genetic screening.

More than half of the participants gathered information and increased their knowledge concerning genetic risk and screening from physicians, genetic counselors, or other professionals (52%). More than 35% of participants declared they did not care about genetic risk/screening information or were not confident with these issues, thus referring substantial illiteracy on this topic. 10.1% collected information from the web and the remaining 2% from other sources (see Figure 4).

Half of the sample (52.8%) had one or more consultations with specialists (gynecologists mainly), family physicians, and geneticists before undergoing genetic exams.

**Figure 5:**
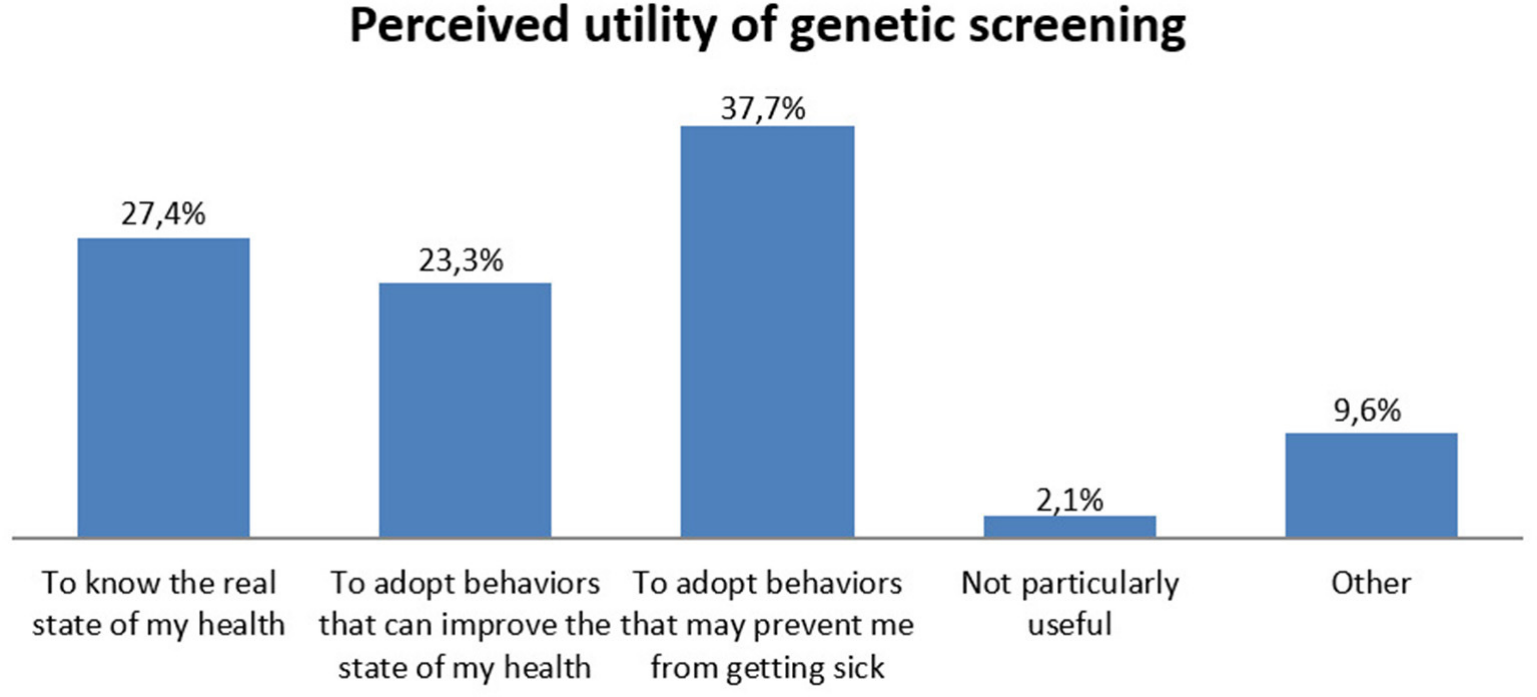
Perceived utility of genetic screening reported by clients.

In Figure 5, results about the perceived utility of genetic testing are represented. Summarizing the first three answers options, the majority of participants thought genetic screening was overall useful (88.4%). In particular, 37.7% perceived genetic screening as useful to adopt behaviors that may prevent disease onset, 27.4% consider it useful to know their “real health status,” and 23.3% consider that genetic screening could help them in adopting health-related behaviors.

**Figure 6:**
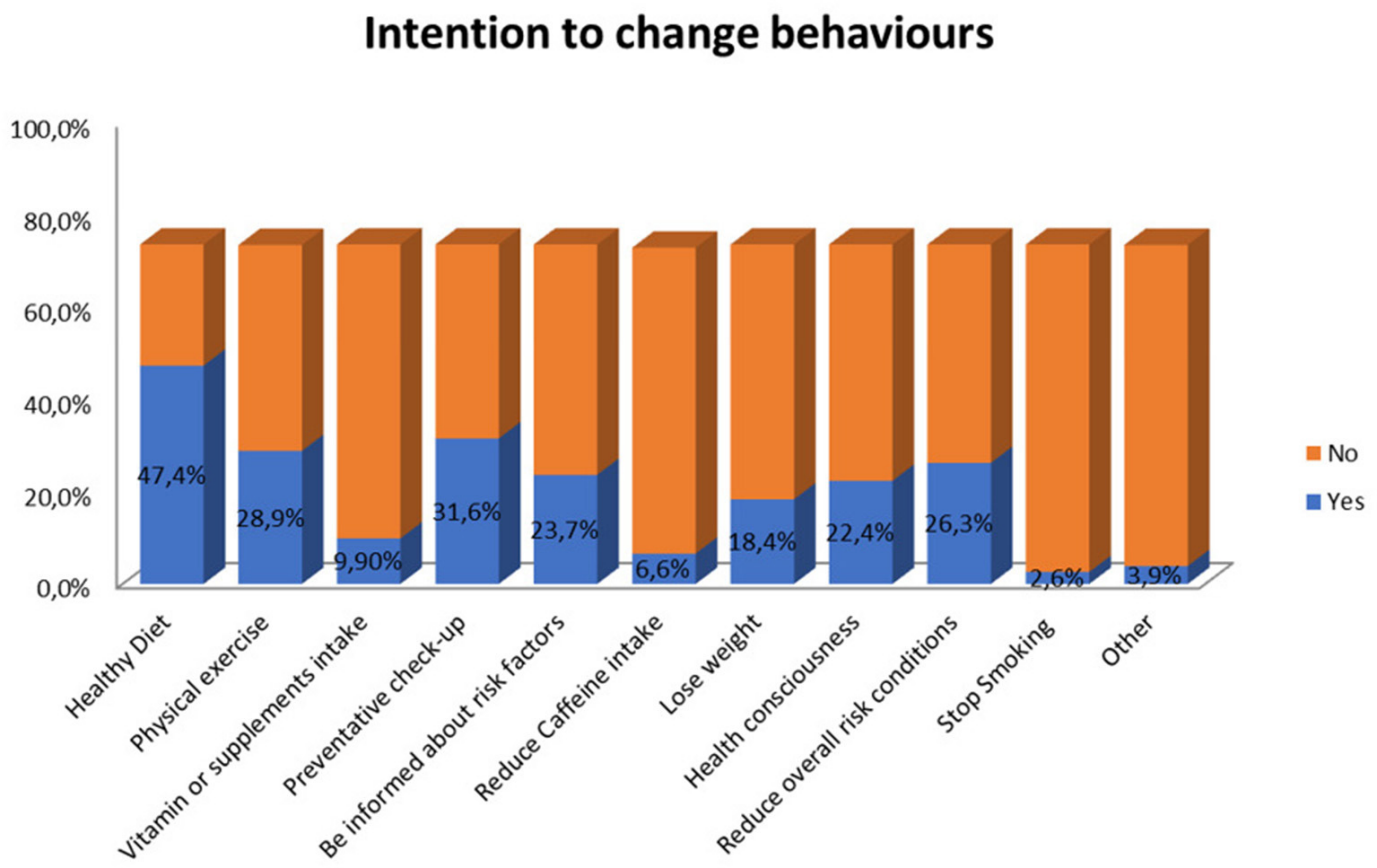
GT users' to change specific behaviours after receiving the genetic test results.

When asked about the intention to change their lifestyle after receiving the genetic test results, 72.3% of participants claimed they were motivated to change their behaviors. Among this population, participants reported the will to follow a healthier diet (47.4%) and undergo preventive medical check-ups (31.6%) (details in Figure 6). 44.8% of the sample stated that they would share the results with their family members, whereas 86.2% would share them with physicians. None declared the intention not to share the results.

### Interaction Among Socio-Demographic Aspects and Decisions Related to Genetic Testing

We investigated the link between parenthood and motivations for taking a genetic test. Pearson Chi-square test showed a significant difference between people who had children vs. people without children [*X*^2^ (*N* = 148) = 24.064, *p* < 0.01] in deciding to undergo genetic testing for procreation purposes or carrier investigation, with participants without children more frequently requiring gene testing for these reasons (43.8% without children vs. 18.7% with children). Differently, participants who had children underwent more frequently genetic testing for cancer susceptibility (8.2% without children vs. 40% with children).

No differences emerged in the level of genetic knowledge and source of information based on the level of education.

### The Link Between the Perceived Utility of Genetic Testing, Knowledge, and Source of Information With Lifestyle Change Intentions

Contingency tables revealed no significant differences between groups based on knowledge/source of information with the perceived utility of genetic testing or changes in lifestyle after results. Participants who reported the intention to change lifestyle after results showed significant differences in the perceived utility of genetic testing, compared to those who declared they would not [*X*^2^ (*N* = 138) = 12.417, *p* = 0.015]. In particular, participants were convinced that genetic testing was useful to “adopt behaviors that can improve the state of my health” (81.8%).

No other significant differences emerged comparing the variables measured.

## Discussion

Considering the recent progress in genetic analysis and the number of people asking for genetic tests, consumer profiling is becoming increasingly important as it is the first step to creating appropriate guidelines for the provision of genetic services, genetic counseling, and education campaigns, and in providing tailored genetic information useful for prevention. Recent evidence showed that public awareness of genomics and personalized medicine was not increasing in line with advancements in the industry (29). In this preliminary study, we investigated the consumers' profiles. Some questions which guided our investigation were the ones solicited by the Center for Disease Control and Prevention, to understand the public health impact of genetic testing provided by private companies (e.g., How the population of genetic testing users is composed in Italy? How many people who participate in DNA tests are motivated by health-related concerns and purposes? How many people believe the results of the test are valuable or helpful? Do they eventually talk to and share their test results with their doctor and or/family members? Do people have any intention to change any of their health behaviors as a result?). Interestingly, our population of Italian consumers was mainly composed of females: there were quadruple the amount of female participants than male. The main reasons to undergo a genetic analysis were to investigate their predisposition to cancer, breast cancer in particular (BRCA 1/2), to understand the cause of fertility problems (e.g., people who underwent genetic testing for celiac disease were sometimes motivated by multiple miscarriages), and to investigate if they were carriers of mutations for specific diseases (and thus discover their risk of hereditary for the future offspring). However, it might be possible that another factor contributes to such a distribution. Indeed, in a recent study which compared German and Italian population of consumers (21), males of both countries answered more frequently that directives for data protection were needed in genetics (70 vs. 50% women) and were more worried than women about “…persons or institutions can control me with the genetic information”: such a deeper privacy concern could contribute to their lower availability in participating in surveys on genetics.

In our sample, participants were predominantly young adults, married and well-educated, white-collar or freelance workers. The well-educated middle class might request genetic tests in private laboratories more than the other categories because they have higher literacy rates and higher economic resources. The potential inequities in the access to genetic information and health care, in general, has been already discussed in the literature (30, 31). For instance, a recent study reported the role of social determinants of health and social identity in creating possible barriers in the access to genetic screening for hereditary breast cancer (32).

Among our participants, those without children were more predisposed to require genetic testing for fertility problems, to prevent genetic transmission to future offspring, or to investigate food intolerances (or for wellness in general); on the contrary, participants with children mainly asked to be tested for cancer predisposition. This result suggests that the condition of being a parent, or the intention to become a parent, influenced the individual's attitude toward genetic testing and the importance given to investigating a predisposition for a threatening disease. Other studies in the literature have shown that subjects who undergo a genetic test for cancer (breast and ovarian) are worried about their offspring and experience decisional conflicts toward their relatives (33, 34).

The main resource of information on genetic tests is the family doctor or the specialist (e.g., gynecologist), who frequently recommended the clients for a specific genetic test. The physician seems to be an important reference even for managing the genetic result; indeed, clients who started their “journey” thanks to the physicians usually decided to continue to trust the physicians after the test for all other communication and for handling the results.

Even if the majority of the participants decided to trust their physicians, a good percentage of clients preferred to receive their result privately and be guided by the services offered in a direct relationship with the genetic company (around 36% maintained direct contact with GenomaLab), thus demonstrating a certain desire for freedom in handling the result and its implications.

Traditionally, genetic tests were the responsibility of health professionals such as doctors, nurses, and genetic counselors (35). The market for direct-to-consumer genetic tests challenged this system and allowed people to autonomously know their genetic make-up for preventive purposes (such as increasing the frequency of medical check-up, adopting healthier lifestyles, or making decisions for themselves and other family members) (36, 37). As reported in a recent contribution, currently there is a wide spectrum of laws regarding genetic testing in Europe (38). Italy and other countries, such as France, Hungary, and Germany, prescribe mandatory medical supervision and restrictions in the way some genetic tests are performed, or in the way pre-symptomatic and susceptibility tests are suggested, allowing them only for healthcare purposes (Italian General Authorization for the Processing of Genetic Data 2014). They also depend on medical prescription for money coverage with the National Health System. However, following our participants' answers, in Italy it seems that there is a shared collaboration between doctor and patient in genetic decision making, and it is an important signal that reaffirms the attribution of confidence to the doctor. The patient assigns the doctor an important role: he/she is the mediator who knows the panorama of available treatments and chooses those most suited to the specific condition of the single patient, even in the genetic setting. It is not a coincidence that, in the Italian context, citizens continue to assign doctors as the main source of information on health issues, recognizing the value of their scientific knowledge, despite the significant increase in the relevance of other information sources, such as television and the internet. If this were true, then future investigations might focus on the level of information of the Italian doctors about genetic matters, and how much they are able to accompany their patients in the decision-making process.

Another interesting aspect emerging from our results was that most clients were used to undergoing regular medical check-ups, followed a Mediterranean diet (vegetables, meat, fish, and pasta), had a mean BMI in the normal range, and practiced moderate or vigorous physical activity, thus depicting a population aware and careful of their health status. Furthermore, the analysis of risky behaviors, such as smoking or damaging food intake, described a population not particularly exposed to the risks assessed in the questionnaire. It thus seems that Italian clients are usually people who tend to behave guided by the need for prevention, and who tend to believe they can have an impact on their health (internal health locus of control). It would be important to investigate how personality characteristics might affect the attitudes in the uptake of genetic risk information (13).

Our population of consumers often complained about sleep disorders and reported current or past experiences with psychological suffering. Despite 62.7% of participants reporting a family history of disease, only a small percentage, 24%, recognize that health problems could be correlated with their family history and thus have a “genetic” origin.

A promising result is that more than half of consumers claim to be motivated to change their behaviors (e.g., healthier diet, practice more exercise or lose weight, undergo preventative medical checks) after testing. Such intentions seem not to depend upon the level of knowledge or the source of information (e.g., physician, internet, family, etc.). These consumers are also convinced that genetic risk analysis is paramount to adopting behaviors that can improve the state of their health. Nevertheless, a recent review showed how behavioral and lifestyle changes depend on people's perception of risk, severity, controllability of the disease, and the availability of treatments (11).

On the other hand, a worrying piece of data shows that a percentage of our population demonstrate a poor sense of agency and self-efficacy in the management of this kind of information: they declared “I do not care about genetic risk/screening information” or “I am not confident with these issues” (35%). This is indicative of a lack of genetic literacy and understanding of how genes work and interact with non-genetic factors, including the environment and lifestyle experiences, in determining disease onset or other clinical conditions (39). Moreover, as observed before, among our participants there have been a similar percentage (36%) of subjects who wanted to independently manage the results with the Lab (without their physician as mediator). These two groups of participants do not overlap; that means not all users who declared to be “illiterate” in genetic matters preferred to manage the results autonomously, and vice versa, not all participants who choose to proceed independently were illiterate, but we must avoid that poorly informed/expert consumers gather complex health information that they would not be able to manage autonomously. In a world where genetic tests influence most people's daily lives, it is fundamental to invest in initiatives that educate the general population on what kind of data are collected, how they can be used, and which conclusions can be drawn from them (40), in order to enhance self-efficacy and empower people in making informed decisions based on genetic information in Europe.

## Conclusion

DTC-GTs for health purposes are now mainstream, and probably will become increasingly common; for this reason, it is paramount to investigate the profiles of genetic companies' clients. Indeed, by knowing their current motivation, beliefs, literacy, and concerns, it will be possible to optimize consumers' experience with GT results and to empower them to make more informed choices. However, several population characteristics (genetic education, attitudes toward one's own health, etc.) may depend upon national education policies and health care systems; therefore, results about GT information impact may vary, also noticeably, in different countries.

This kind of survey contributes to building a set of easily accessible information about DTC-GT users in different EU nations; also, they allow to take steps for empowering consumers to make more informed choices about their behavior and resource expenditures for health.

These measures could include providing:

- Education focused on gaps in public genetic literacy and knowledge;
- Fine-grained tailored communications about specific test characteristics and their advantages/disadvantages to different profiles of DTC-GT clients;
- More detailed information about data protection in order to overcome the more common privacy concerns.

## Limits and Further Researches

We recognize some limitations for the current investigation. The sample of 152 Italian GT consumers is quite small, so the results could not be generalized outside of this specific context, and further research is needed to amplify the significance of what has been observed. However, the population of GT clients recruited in this study underwent a further investigation of their psychological profile (41) and two follow-ups, at 6 months and 1 year after the receipt of the result, to monitor the actual changes and decisions made over time. Currently, the data are being processed and on the point of being considered for publication. Another limitation is that our population was mostly healthy, and we don't have data about people that had a specific full-blown pathology, who might have made different choices in this particular scenario. Moreover, our population was very specific as it was enrolled in a single GT service/laboratory. So further studies are needed to compare populations from different private GT laboratories in Italy and in Europe.

## Data Availability Statement

The datasets generated for this study are available on request to the corresponding author.

## Ethics Statement

The studies involving human participants were reviewed and approved by Institutional Review Board of the University of Milan, the principal coordination center of the survey, and by the Center for Research Ethics and Bioethics, University of Uppsala, coordinator of the Mind the Risk project. The patients/participants provided their written informed consent to participate in this study.

## Author Contributions

SO collected data and wrote the main part of the manuscript. FS collected data and provided revisions. GM and LV provided the analysis and wrote the results section. IC and AG provided revisions and comments. GP supervised the work and contributed to discussion and conclusions section. All authors contributed to the article and approved the submitted version.

## Conflict of Interest

FS was employed by the company GenomaLab. The remaining authors declare that the research was conducted in the absence of any commercial or financial relationships that could be construed as a potential conflict of interest.

## Abbreviations

DTC-GT: Direct to Consumer genetic testing
BMI: Body Max Index
IPAQ-SF: International Physical Activity Questionnaire–Short Form.

## References

[1] De Rosa S Arcidiacono B Chiefari E Brunetti A Indolfi C Foti DP. Type 2 diabetes mellitus and cardiovascular disease: genetic and epigenetic links. Front Endocrinol. (2018) 9:2. 10.3389/fendo.2018.00002 29387042 PMC5776102

[2] Foulkes WD. Inherited susceptibility to common cancers. N Engl J Med. (2008) 359:2143–53. 10.1056/NEJMra0802968 19005198

[3] Grosse SD Khoury MJ. What is the clinical utility of genetic testing? Genet Med. (2006) 8:448–50. 10.1097/01.gim.0000227935.26763.c6 16845278

[4] Herceg Z. Epigenetics and cancer: towards an evaluation of the impact of environmental and dietary factors. Mutagenesis. (2007) 22:91–103. 10.1093/mutage/gel068 17284773

[5] Portela A Esteller M. Epigenetic modifications and human disease. Nat Biotechnol. (2010) 28:1057–68. 10.1038/nbt.1685 20944598

[6] Sharma S Kelly TK Jones PA Epigenetics in cancer. Carcinogenesis. (2010) 31:27–36. 10.1093/carcin/bgp220 19752007 PMC2802667

[7] Resnik DB Genetics and personal responsibility for health. New Genet Soc. (2014) 33:113–25. 10.1080/14636778.2014.905195 25598702 PMC4293629

[8] Oliveri S Pravettoni G Fioretti C Hansson MG Let the individuals directly concerned decide: a solution to tragic choices in genetic risk information. Public Health Genomics. (2016) 19:307–13. 10.1159/000448913 27603671

[9] Renzi C Riva S Masiero M Pravettoni G. The choice dilemma in chronic hematological conditions: why choosing is not only a medical issue? A psycho-cognitive perspective. Crit Rev Oncol Hematol. (2016) 99:134–40. 10.1016/j.critrevonc.2015.12.010 26762858

[10] Marton G Pizzoli SFM Vergani L Mazzocco K Monzani D Bailo L Patients' health locus of control and preferences about the role that they want to play in the medical decision-making process. Psychol Health Med. (2020) 23:1–7. 10.1080/13548506.2020.1748211 32323553

[11] Oliveri S Ferrari F Manfrinati A Pravettoni G A systematic review of the psychological implications of genetic testing: a comparative analysis among cardiovascular, neurodegenerative and cancer diseases. Front Genet. (2018) 9:624 10.3389/fgene.2018.00624 30619456 PMC6295518

[12] Oliveri S Masiero M Arnaboldi P Cutica I Fioretti C Pravettoni G. Health orientation, knowledge, and attitudes toward genetic testing and personalized genomic services: preliminary data from an Italian sample. Biomed Res Int. (2016) 2016:1–9. 10.1155/2016/6824581 28105428 PMC5220460

[13] Mavroidopoulou V Xera E Mollaki V Awareness, attitudes and perspectives of direct-to-consumer genetic testing in Greece: a survey of potential consumers. J Hum Genet. (2015) 60:515–23. 10.1038/jhg.2015.58 26040209

[14] Mai Y Koromila T Sagia A Cooper DN Vlachopoulos G Lagoumintzis G. A critical view of the general public's awareness and physicians' opinion of the trends and potential pitfalls of genetic testing in Greece. Per Med. (2011) 8:551–61. 10.2217/pme.11.48 29793257

[15] Vermeulen E Henneman L van El CG Cornel MC. Public attitudes towards preventive genomics and personal interest in genetic testing to prevent disease: a survey study. Eur J Public Health. (2014) 24:768–75. 10.1093/eurpub/ckt143 24068545

[16] Berth H Balck F Dinkel A. Attitudes toward genetic testing in patients at risk for HNPCC/FAP and the German population. Genet Test. (2002) 6:273–80. 10.1089/10906570260471804 12537651

[17] Vayena E Ineichen C Stoupka E Hafen E Playing a part in research? University Students' attitudes to direct-to-consumer genomics. Public Health Genomics. (2014) 17:158–68. 10.1159/000360257 24777115

[18] Charbonneau J Nicol D Chalmers D Kato K Yamamoto N Walshe J Public reactions to direct-to-consumer genetic health tests: a comparison across the US, UK, Japan and Australia. Eur J Hum Genet. (2020) 28:339–48. 10.1038/s41431-019-0529-8 31645768 PMC7029038

[19] Covolo L Rubinelli S Ceretti E Gelatti U. Internet-based direct-to-consumer genetic testing: a systematic review. J Med Internet Res. (2015) 17:e279. 10.2196/jmir.4378 26677835 PMC4704942

[20] Su Y Howard HC Borry P. Users' motivations to purchase direct-to-consumer genome-wide testing: an exploratory study of personal stories. J Community Genet. (2011) 2:135–46. 10.1007/s12687-011-0048-y 22109820 PMC3186033

[21] Wöhlke S Schaper M Oliveri S Cutica I Spinella F Pravettoni G. German and Italian users of web-accessed genetic data: attitudes on personal utility and personal sharing preferences. results of a comparative survey (n=192). Front Genet. (2020) 11:102. 10.3389/fgene.2020.00102 32265977 PMC7099127

[22] Rafiq M Ianuale C Ricciardi W Boccia S. Direct-to-consumer genetic testing: a systematic review of european guidelines, recommendations, and position statements. Genet Test Mol Biomarkers. (2015) 19:535–47. 10.1089/gtmb.2015.0051 26313927

[23] Borry P van Hellemondt RE Sprumont D Jales CFD Rial-Sebbag E Spranger TM. Legislation on direct-to-consumer genetic testing in seven European countries. Eur J Hum Genet. (2012) 20:715–21. 10.1038/ejhg.2011.278 22274578 PMC3376265

[24] Oliveri S Pravettoni G The disclosure of direct-to-consumer genetic testing: sounding out the psychological perspective of consumers. Biol Med. (2016) 8:5 10.4172/0974-8369.1000316

[25] Oliveri S Renzi C Pravettoni G. Toward an in-depth profiling of DTC users. Clin Genet. (2015) 88:505–6. 10.1111/cge.12599 25959657

[26] Oliveri S Pravettoni G Capturing how individuals perceive genetic risk information: a phenomenological perspective. J Risk Res. (2018) 21:259–67. 10.1080/13669877.2017.1281333

[27] Oliveri S Renzi C Masiero M Pravettoni G. Living at risk: factors that affect the experience of direct-to-consumer genetic testing. Mayo Clin Proc. (2015) 90:1323–6. 10.1016/j.mayocp.2015.06.014 26434959

[28] Lee PH Macfarlane DJ Lam T Stewart SM. Validity of the international physical activity questionnaire short form (IPAQ-SF): a systematic review. Int J Behav Nutr Phys Act. (2011) 8:115. 10.1186/1479-5868-8-115 22018588 PMC3214824

[29] Krakow M Ratcliff CL Hesse BW Greenberg-Worisek AJ. Assessing genetic literacy awareness and knowledge gaps in the US population: results from the health information national trends survey. Public Health Genomics. (2017) 20:343–8. 10.1159/000489117 29852491 PMC6095736

[30] McClellan KA Avard D Simard J Knoppers BM. Personalized medicine and access to health care: potential for inequitable access? Eur J Hum Genet. (2013) 21:143–7. 10.1038/ejhg.2012.149 22781088 PMC3548263

[31] Smith CE Fullerton SM Dookeran KA Hampel H Tin A Maruthur NM. Using Genetic technologies to reduce, rather than widen, health disparities. Health Aff . (2016) 35:1367–73. 10.1377/hlthaff.2015.1476 27503959 PMC5100696

[32] Sayani A. Inequities in genetic testing for hereditary breast cancer: implications for public health practice. J Community Genet. (2019) 10:35–9. 10.1007/s12687-018-0370-8 29781042 PMC6325036

[33] Rini C O'Neill SC Valdimarsdottir H Goldsmith RE Jandorf L Brown K. Cognitive and emotional factors predicting decisional conflict among high-risk breast cancer survivors who receive uninformative BRCA1/2 results. Health Psychol. (2009) 28:569–78. 10.1037/a0015205 19751083 PMC3510002

[34] Katapodi MC Northouse L Pierce P Milliron KJ Liu G Merajver SD. Differences between women who pursued genetic testing for hereditary breast and ovarian cancer and their at-risk relatives who did not. Oncol Nurs Forum. (2011) 38:572–81. 10.1188/11.ONF.572-581 21875844

[35] Ormond KE Laurino MY Barlow-Stewart K Wessels T Macaulay S Austin J. Genetic counseling globally: where are we now? Am J Med Genet Part C Semin Med Genet. (2018) 178:98–107. 10.1002/ajmg.c.31607 29575600 PMC5947883

[36] Kaput J Nutrigenomics research for personalized nutrition and medicine. Curr Opin Biotechnol. (2008) 19:110–20. 10.1016/j.copbio.2008.02.005 18387295

[37] Guttmacher AE Collins FS. Welcome to the Genomic Era. N Engl J Med. (2003) 349:996–8. 10.1056/NEJMe038132 12954750

[38] Kalokairinou L Howard HC Slokenberga S Fisher E Flatscher-Thöni M Hartlev M. Legislation of direct-to-consumer genetic testing in Europe: a fragmented regulatory landscape. J Community Genet. (2018) 9:117–32. 10.1007/s12687-017-0344-2 29150824 PMC5849704

[39] Kampourakis K Public understanding of genetic testing and obstacles to genetics literacy. In: *Molecular Diagnostics*. London: Academic Press; Elsevier 2017. p. 469–477. 10.1016/B978-0-12-802971-8.00027-4

[40] Mainetti R Oliveri S Cutica I Gorini A Gaspardo S Borghese NA Design, development and usability test of serious games related to genetics. In: *2018 IEEE 6th International Conference on Serious Games and Applications for Health (SeGAH)*. Vienna: IEEE p. 1–8. 10.1109/SeGAH.2018.8401344

[41] Oliveri S Durosini I Cutica I Cincidda C Spinella F Baldi M. Health orientation and individual tendencies of a sample of Italian genetic testing consumers. Mol Genet Genomic Med. (2020) 8:e1291. 10.1002/mgg3.1291 32500972 PMC7434739

